# Editorial: Ferroptosis in cancer and beyond

**DOI:** 10.3389/fmolb.2022.1115974

**Published:** 2023-01-04

**Authors:** Xin Wang, Guo Chen, Chaoyun Pan, Yanqing Liu

**Affiliations:** ^1^ National Institute of Neurological Disorders and Stroke, Bethesda, MD, United States; ^2^ School of Biopharmacy, China Pharmaceutical University, Nanjing, China; ^3^ Department of Biochemistry and Molecular Biology, Zhongshan School of Medicine, Sun Yat-sen University, Guangzhou, China; ^4^ Herbert Irving Comprehensive Cancer Center, Columbia University, New York, NY, United States

**Keywords:** ferroptosis, oxidative stress, cancer, neurodegenerative disease, organ injury, disease therapy

## Introduction

To die or not to die, that’s not a question for eukaryotic cells. The issues that really matter are WHEN and HOW to die. Cell death has been observed for quite a long time. Historically, this process was taken for granted as passive and uncontrolled, until the discovery of regulated cell death (RCD) (Galluzzi et al., 2018). RCD is a generic term referring to cell death types that can be modulated genetically or pharmacologically, which is contrast to accidental cell death (ACD, which is caused by chemical, physical, or mechanical stresses and cannot be regulated) (Galluzzi et al., 2018). RCD is an evolutionary strategy for both unicellular and multicellular lives to orchestrate normal development and growth (programmed cell death, PCD) or cope with extracellular or intracellular insults (stress-drived RCD) (Galluzzi et al., 2016). Over a long period, RCD and PCD were synonymous to apoptosis—the first discovered RCD type (Hengartner, 2000). In the last two decades, many more types of novel RCDs were identified, such as necroptosis (Vandenabeele et al., 2010), pyroptosis (Shi et al., 2017), and ferroptosis (Stockwell et al., 2017; Dixon and Stockwell, 2019). According to the Nomenclature Committee on Cell Death (NCDD), to date there are more than ten RCD modalities (Galluzzi et al., 2018). All of these cell death processes are featured by specific cellular morphology, molecular mechanism and physiological/pathological significance, while they may also share some similarities in these aspects. Among them, ferroptosis stands out as one of the most interesting Research Topics in the RCD field and shows critical roles in development along with great therapeutic potential for various pathological disorders (Stockwell et al., 2017; Dixon and Stockwell, 2019).

Ferroptosis is caused by the unchecked oxidative perturbation within the cell. Since its discovery, ferroptosis field has caught much attention for not only its significance in basic life sciences as a novel RCD, but also its great potential in clinical use. Actually, many disorders have been linked to ferroptosis (Qiu et al., 2020; Stockwell et al., 2020), such as cancer (Hassannia et al., 2019), ischemia-reperfusion injury (Pefanis et al., 2019), and neurodegenerative disease (Ratan, 2020). Although some molecular mechanisms for the initiation and regulation of ferroptosis have been dug out, there are still many critical questions await to be addressed, along with more and more emerging new research results that are refreshing our knowledge about the role of ferroptosis in cancer and other diverse disorders. Based on whatever purpose, scientifically or clinically, this field deserves much more devotion to harvest more fruitful achievements. To summarize and advance the development of this field, we organized this Research Topic titled “*Ferroptosis in cancer and beyond*”, aiming to report on the basic mechanisms underlying ferroptosis, how ferroptosis is regulated, and how ferroptosis pathways can be targeted therapeutically in various diseases, particularly in cancer. This Research Topic has shown great success, as we have collected 33 papers in total. These papers include cutting-edge research articles and reviews about the regulation and pathological function of ferroptosis. We would like to briefly introduce these papers below.

### Regulation of ferroptosis

Ferroptosis is delicately regulated, with many factors and pathways involved in its initiation, propagation, and termination (Dixon and Stockwell, 2019; Liu and Gu, 2022a). Among them, GPX4 and p53 are the two core regulators of ferroptosis (Liu and Gu, 2022a; Liu and Gu, 2022b). Cui et al. discussed how post-translational protein modification modulates GPX4 expression and activity, thereby influencing its role to antagonize ferroptosis. For p53, the review by Zhang et al. focuses on the potential crosstalk between p53 and G-quadruplex of DNA or RNA, adding new mechanism to the regulation of p53 function in ferroptosis (Liu et al., 2019). SLC7A11 is a repressive target of p53, and also an upstream regulator of GPX4, therefore linking p53-and GPX4-mediated ferroptosis pathways (Jiang et al., 2015). Cheng et al. found that SLC7A11 is upregulated in colon adenocarcinoma than in normal colon tissues and is associated with various tumor hallmarks of colon adenocarcinoma. SLC3A2 and SLC7A11 form a complex called System Xc− to import cystine into the cell. He et al. analyzed the expression of SLC3A2 across different tumor types and investigated its correlation with tumor microenvironment, especially the immune cell function. Autophagy-dependent ferritinophagy contributes to ferroptosis initiation. Liang et al. discovered that NPC intracellular cholesterol transporter 1 (NPC1) has a role in regulating ferritinophagy, ferroptosis, and hearing loss. MicroRNA is a type of pleiotropic small RNA to regulate various biological processes, including cancer (Liu et al., 2016; Liu et al., 2017; Liu et al., 2018; Ma et al., 2019). In the review by Guo et al. authors introduced how different microRNAs participate in the regulation of ferroptosis. In addition, Liu et al. used single-cell transcriptomics technology to identify many key regulators of ferroptosis in head and neck squamous cell carcinoma.

### Ferroptosis and tumor

Ferroptosis is associated with the development of many diseases, with tumor as a notable example. In fact, till now there are numerous papers reporting that ferroptosis involves the transformation processes of distinct tumor types. In this Research Topic, Qu et al. wrote a good review to summarize the role of ferroptosis in different cancers. They firstly introduced the basic mechanism and regulation of ferroptosis. Then, they discussed how ferroptosis participates in the regulation of tumor development. They also touched the therapeutic potential by targeting ferroptosis in tumors, with a summary of the available ferroptosis inducers**.** Maimaitizunong et al. contributed a comprehensive review about ferroptosis and esophagus cancer. In their paper, the regulators and signaling pathways of ferroptosis are discussed in detail. They also depicted various applications related to ferroptosis in esophagus cancer. The figures and tables in this paper are well-presented and informative. In addition, Fan et al. focused on ferroptosis and gynecological malignancies. Due to the significant role of ferroptosis, it is obvious that targeting ferroptosis can be an effective way to treat cancer. Tan et al. proposed a question: is ferroptosis enemy or friend in tumor therapy? On one side, they thought targeting ferroptosis may be good to many tumor patients. On the other side, they warned the researchers to take possible side effect of inducing ferroptosis into consideration when treating cancers. Indocyanine green is a near-infrared fluorescent molecule used in the imaging of residual tumor removal during surgery. A study by Tseng et al. found that OATP1B3-mediated uptake of indocyanine green facilitated ferroptosis when exposed to 808-nm laser irradiation in HT1080 cell. This result is consolidated by *in vivo* data. These results suggested that indocyanine green plus laser radiation may be a new treatment method for chemo-resistant cancers by inducing ferroptosis. To identify efficient agents to target ferroptosis in non-small cell lung cancer, Zhao et al. used bioinformatics to screen lead compounds that induce ferroptosis in tumor cells. By doing so, they identified flufenamic acid could promote ferroptosis and suppress growth and migration in A549 cell line. Moreover, three excellent reviews by Zhuo et al., Wu et al., and Li et al. discussed the therapeutic potential by modulating ferroptosis in glioblastoma, endometrial cancer, and hepatocellular carcinoma, respectively. Beyond as treatment target, ferroptosis-related genes can also have prognostic potential in cancer and other disorders (Liu et al., 2022; Ye et al., 2022). In hepatocellular carcinoma, Zhang et al. analyzed the differentially expressed ferroptosis-related genes by using the TCGA database. Based on the analysis result, they constructed a gene signature that is associated with immune cell infiltration in hepatocellular carcinoma. Importantly, their signature can effectively reflect the prognosis of hepatocellular carcinoma patients. In addition, this model also works well when considering other different characteristics of hepatocellular carcinoma. This study flow encourages the mining of prognostic usage of ferroptosis-related gene signatures in other tumor types. Indeed, by using similar approaches, Tian et al., Yin et al., and Hu et al. expanded the prognostic potential of ferroptosis to glioblastoma, acute myeloid leukemia, and colorectal cancer, respectively. What should not be missed is Tao et al.’s result. Their ferroptosis-related mRNAs and lncRNAs model is a wonderful supplement to the classic 2017 European LeukemiaNet (ELN) classification system of pediatric acute myeloid leukemia.

### Ferroptosis and other diseases

Unlike its tumor-suppressive role that can be leveraged in tumor treatment, ferroptosis is often one of the causes in neurodegenerative diseases and organ injury, which should be avoided. In this Research Topic, there is an excellent review by Ma et al. to summarize connection between Alzheimer’s disease and the ferroptosis signaling pathways. To begin with, the authors introduced the major regulators and signaling pathways in ferroptosis. Next, they introduced in detail the link between these factors (including iron metabolism, lipid metabolism, amino acid metabolism, and GPX4) with Alzheimer’s disease. Then, they suggested that ferroptosis inhibitors could be used to treat Alzheimer’s disease. In Parkinson’s disease, Wang et al. found that increased iron deposition in nigrosome might be a critical pathological cause for Parkinson’s disease. Mechanistically, iron deposition may increase the sensitivity of neuron to ferroptosis. Development of iron deposit modulation drug is a practical way to prevent and treat Parkinson’s disease. Ferroptosis in brain may also happen after intracerebral hemorrhage. Ren et al. in their review discussed the risk of brain injury after intracerebral hemorrhage. They concluded that the induction of ferroptosis is a risk factor for after intracerebral hemorrhage-related brain injury and should be monitored and inhibited in clinical practice. In kidney, the occurrence of ferroptosis is associated with acute kidney injury and renal fibrosis. In Zhang et al.’s and Zhang et al.’s reviews respectively, the readers can find detailed information how ferroptosis contribute to these kidney disorders and how to prevent it. Beyond brain and kidney, ferroptosis also functions in liver, lung, heart, and skeletal muscle. Zhou et al. concluded the effect of ferroptosis in multiple chronic liver diseases, including alcoholic liver disease, non-alcoholic fatty liver disease, liver fibrosis, hepatocellular carcinoma. Remarkably, they proposed some limitations in the current ferroptosis studies in chronic liver diseases. Li et al. discussed extensively the contribution of ferroptosis to the pathologic process of lung diseases, such as obstructive lung diseases (chronic obstructive pulmonary disease, asthma, and cystic fibrosis), interstitial lung diseases (pulmonary fibrosis of different causes), pulmonary diseases of vascular origin (ischemia-reperfusion injury and pulmonary hypertension), pulmonary infections (bacteria, viruses, and fungi), acute lung injury, acute respiratory distress syndrome, obstructive sleep apnea, pulmonary alveolar proteinosis, and lung cancer. In another review, Lin et al. focused on ferroptosis and airway inflammatory diseases. Noteworthily, they also discussed the potential link between ferroptosis and COVID-19 infection. Ageing is along with cardiac dysfunction. Liang et al. found that ageing-associated ferroptosis is a reason for this process. In addition, hydrogen sulfide was demonstrated to protect aging rat from cardiac dysfunction by suppressing cardiomyocyte ferroptosis. Wang et al.’s review reports the correlation between ferroptosis and various skeletal muscle diseases, including sarcopenia, rhabdomyolysis, rhabdomyosarcoma, exhaustive exercise-induced fatigue, and idiopathic inflammatory myopathy. Ferroptosis even has a role in psoriasis. The review by Zhou et al. depicts the pathogenesis of psoriasis, the mechanisms of ferroptosis, and the connection between these two processes. Moreover, authors also discussed the therapeutic potential of ferroptosis inhibitors in psoriasis.

## Conclusion and perspective

To sum up, this Research Topic covers a batch of up-to-date papers about ferroptosis research, with an emphasis on the role of ferroptosis in tumor and other multiple diseases. We believe our Research Topic could provide invaluable information to the readers interested in ferroptosis field. We also look forward more exciting and important research results could come out in the near future.
